# Comprehensive Analysis of Annexin Family from *Tetragonia tetragonoides* and Its Initial Functions in Abiotic Stress Responses

**DOI:** 10.3390/plants15142218

**Published:** 2026-07-21

**Authors:** Lihua Chen, Fuying Xie, Shuguang Jian, Zhengfeng Wang, Tingyao Li, Mei Zhang

**Affiliations:** 1 Center of Economic Botany, Core Botanical Gardens; Guangdong Provincial Key Laboratory of Applied Botany, South China Botanical Garden, Chinese Academy of Sciences, Guangzhou 510650, China; 2 University of the Chinese Academy of Sciences, Beijing 100039, China; 3 Southern Marine Science and Engineering Guangdong Laboratory (Guangzhou), Guangzhou 511458, China; 4 Vegetable Research Institute, Guangdong Academy of Agricultural Sciences, Guangzhou 510640, China

**Keywords:** annexin, abiotic stress tolerance, gene expression, *Tetragonia tetragonoides*

## Abstract

*Tetragonia tetragonoides* is a valuable medicinal and nutritional wild plant and a typical tropical euhalophyte, exhibiting strong tolerance to salinity, alkalinity, drought, and heat. Plant annexins play critical roles in plant growth, development, and stress responses; however, the characteristics and stress-responsive patterns of the annexin (*TtAnn*) gene family in *T. tetragonoides* remain largely unclear. This study aimed to systematically characterize the TtAnn family and explore its functions in abiotic stress tolerance. A total of 15 *TtAnn* members were identified at the genome-wide level, followed by phylogenetic classification, chromosomal distribution analysis, duplication pattern characterization, *cis*-acting element prediction, and organ-specific as well as stress-induced expression profiling. Five *TtAnn* genes were further cloned and heterologously expressed in yeast for stress tolerance validation. The 15 *TtAnns* were classified into four subgroups and unevenly distributed across nine chromosomes, with whole-genome duplication serving as the predominant mechanism driving gene expansion. Their promoter regions contained multiple *cis*-elements related to light, hormones, and transcription factors. *TtAnns* exhibited root-preferential expression and showed distinct differential expression patterns under salt, alkali, osmotic, and heat stresses. Transgenic yeast assays further confirmed their roles in stress resistance. This study elucidates the fundamental features of the *TtAnn* family and provides valuable gene resources and a theoretical basis for dissecting abiotic stress mechanisms and genetically improving stress-tolerant crops.

## 1. Introduction

Annexins (Anns) are a group of ubiquitous, evolutionarily conserved Ca^2+^-dependent lipid-binding small proteins (32–36 kDa) present in plants, animals, and microorganisms [1,2]. Anns are a family of abundant (0.1% of total cell protein) and highly conserved small water-soluble proteins in plant cells [3]. Anns are found in almost every species, including plants, fungi, vertebrates, invertebrates, protists, and prokaryotes. The first plant Ann was isolated from in vitro tomato cell in 1989 [4]. The plant Ann family has gradually attracted more attention in recent years due to the availability of whole-genome sequencing [2,5]. This interest stems from its role as core regulator or effector in plant growth and various abiotic/biotic stress responses via Ca^2+^-mediated signaling [6].

Plant Anns contain a relatively short N-terminal region and one to four conserved Ann domains in the C-terminus. Their N-terminal sequences often show high variability among plant species [2,7]. The C-terminal core Ann domains usually comprise 70 amino acids with a four-fold repeat (I–IV); each repeat forms the characteristic five-helix bundle with membrane binding sites situated in different loops [1,8]. Due to the specific loops of Ann-conforming channels in the cellular membrane, plant Anns have been identified as membrane transporters for calcium ions (Ca^2+^) in addition to performing other biological functions [9]. Additionally, sequence analysis and gene function identification assays have shown that plant Anns possess peroxidase and ATPase/GTPase activities. They also play roles in actin-binding and are found within the endomembrane and plasma membrane, where they act as unconventional transporters [10,11]. Therefore, Anns play a crucial role in plant stress resistance. However, current research regarding their functions in special habitat-specific plants, especially in halophytes, is limited. Analyzing their functions could further enhance our understanding of the importance of the *Ann* gene family in plant abiotic stress responses.

The roles of plant *Anns* in abiotic stress responses have been intensively investigated, with particular focus on *Anns* in Arabidopsis (*AtAnns*) [2]. *AtAnns* exhibit gene-specific differential expression patterns during development and abiotic stress responses [12,13]. Previous studies indicate that AtAnn1 functions as a multifunctional protein with peroxidase activity, mediating signals in response to drought, salt, heat, and heavy metal stress [6,14,15,16,17]. *AtAnn1*’s expression was also induced by salicylic acid (SA), implicating that this Ann may be involved in pathogen defense responses [18]. Suppression or overexpression of *AtAnn1* resulted in sensitivity or resistance to the plant parasitic nematode, *Meloidogyne incognita* [19]. In addition, *AtAnn1* and *AtAnn2* double mutant *ann1ann2* showed more susceptibility to the plant pathogen *Botrytis cinerea* in comparison to the wild type [20]. These findings further proved that *AtAnn1* positively regulated plant biotic and abiotic stress responses. Furthermore, other *AtAnns*, including *AtAnn2*, *AtAnn3*, *AtAnn4*, and *AtAnn8*, have successively shown multiple tolerances to stress treatments [2,6]. Overall, research into the mechanisms of *AtAnns* in growth, development, and stress responses has provided a solid foundation for studying the *Ann* gene family in other plant species.

Some research has demonstrated that plant Anns participate in cellular adaptation to adverse environments, acting as central regulators of plant growth and stress signaling [6,8,21]. In recent years, the advancement of plant genome sequencing has facilitated the genomic characterization of wild plants and non-essential crops, enabling a deeper exploration of the role of Anns in stress tolerance. The Ann family members have been identified in many plant species, including pepper (*Capsicum annuum*) [5], wheat (*Triticum aestivum*) [22], three Brassicaceae species [23], radish (*Raphanus sativus*) [24], rye (*Secale cereale*) [25], poplar (*Populus trichocarpa*) [26], *Schrenkiella parvula* and *Eutrema salsugineum* [27], chickpea (*Cicer arietinum*) [28], crape myrtle (*Lagerstroemia indica*) [29], cotton (*Gossypium hirsutum*) [30], grape (*Vitis vinifera*) [31], Yanhuanglian (*Corydalis saxicola*) [32], and maize (*Zea mays*) [33]. Exploration for the biological functions of these Ann families was carried out initially, mainly based on transcription analysis and transgenic over-expression testing for specific *Ann* genes. In general, most identified members of the Ann family have been shown to effectively mediate multiple abiotic stress responses, including salt, drought, heat, and oxidative stresses.

Specific plant *Ann* genes with potential roles in stress tolerance have also garnered significant interest, particularly those identified in wild species from special habitats. It is particularly worth noting that in Arabidopsis and its two related halophytic species, *Schrenkiella parvula* and *Eutrema salsugineum*, the gene numbers and sequence identities of *AtAnn*, *SpAnn*, and *EsAnn* all showed some differences [27]. As extremophytes, *S. parvula* and *E. salsugineum* can survive in harsh environments, including dry, salty, or cold conditions, while the glycophyte *Arabidopsis thaliana* is sensitive to high salinity and drought. The comparative study of the *Ann* gene family in these three Brassicaceae species further clarified the possible roles that the *Ann* gene family contributes to the halophyte’s salt tolerance. The regulatory mechanisms for divergent expression patterns of the *Anns* need further investigation to make clear the roles of candidate *Ann* genes in abiotic stress responses.

In this study, a global survey of the 15 *Ann* genes in the *Tetragonia tetragonoides* (Pall.) Kuntze genome was conducted. *T. tetragonoides* (also called New Zealand spinach or French spinach) is an edible plant that has good tolerance to salt, drought, and barren soil [34]; thus, this species shows great potential for ecological restoration of coastal areas and islands. Therefore, understanding the molecular adaptation mechanism of *T. tetragonoides* to its native habitats appears to be particularly important for research on plant stress resistance. In this study, the physicochemical properties and conserved motifs of *TtAnn* family proteins, gene structures, and *cis*-elements (CEs) in the promoter regions of *TtAnn* genes were identified. In addition, the *TtAnn* genes’ homology was determined by collinearity analysis. Furthermore, the transcript analysis by RNA sequencing (RNA-seq) and quantitative reverse transcription PCR (qRT-PCR) results demonstrated that the *TtAnn* transcripts displayed organ-specific expression patterns across various tissues and showed up- or down-regulated patterns under different abiotic stress challenges. The over-expression assay with a yeast system indicated that *TtAnns* could affect the tolerance of different yeast strains to multiple stress challenges. In summary, the present study provides insight into the characterizations and structures of *TtAnns*. These findings lay the foundation for elucidating their biological function in response to abiotic stress, as well as the adaptive mechanisms of *T. tetragonoides* to extreme adversity habitats.

## 2. Results

### 2.1. Identification of Ann Genes from T. tetragonoidese

To identify the *T. tetragonoides* annexin (TtAnn) family, a protein BLAST (https://blast.ncbi.nlm.nih.gov/Blast.cgi, accessed on 29 October 2025) search was performed against the genome-wide protein sequences, resulting in the identification of 15 *TtAnn* candidates. All members were then verified for the presence of Ann repeats using InterPro and the Conserved Domain Database (CDD) of the National Center for Biotechnology Information (NCBI). The corresponding genes were then named as *TtAnn1*–*15*, based on their distribution on the total 16 chromosomes of *T. tetragonoides*.

The amino acid length of the TtAnn proteins ranged from 244 aa (TtAnn10) to 344 aa (TtAnn6); the molecular weights ranged from 28.41 kDa (TtAnn10) to 38.9 kDa (TtAnn6); and the theoretical isoelectric points (PIs) ranged from 5.68 (TtAnn14) to 9.11 (TtAnn4, TtAnn8, and TtAnn9) (Table 1). Regarding the amino acid composition, leucine (L), glutamate (E), lysine (K), and alanine (A) were the predominant residues in the TtAnns, which might reflect the characteristics and biochemical functions of the proteins. In addition, the instability indices (IIs) were relatively low, with only TtAnn3, TtAnn4, TtAnn6, TtAnn10, and TtAnn13 exhibiting unstable characteristics (II > 40). The aliphatic indices (AIs) of all TtAnns were relatively high, and the Grand Average of Hydropathicities (GRAVYs) of all of the TtAnns were negative values, indicating that these proteins are hydrophilic. TransMembrane prediction using Hidden Markov Models (TMHMM) prediction indicated that all of the TtAnns held no transmembrane helices, and subcellular localization prediction by WoLF_PSORT (an extension of the Protein Subcellular Localization Prediction Tool) and the ensemble classifier Plant-PLoc also indicated that most of the *TtAnns* (except *TtAnn8*) were cytoplasmic proteins (Table 1). Thus, these TtAnns were speculated to play biochemical roles in the cytoplasm.

### 2.2. TtAnn Gene Structures, Chromosomal Localization, and Collinearity Analysis of TtAnns

**Figure 1:**
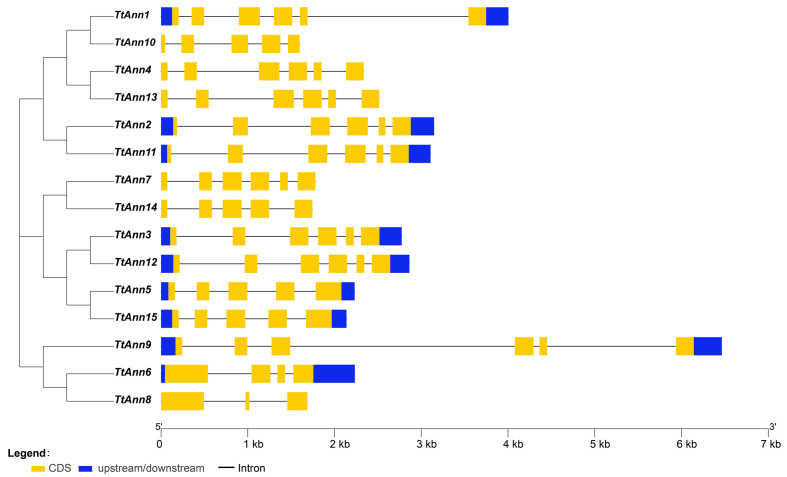
The gene structures of the *TtAnns*. The left part shows the phylogenetic relationships of the cDNAs of 15 *TtAnns* from *T. tetragonoides* genome sequences.

The *TtAnn* gene family structures were analyzed and drawn using the Gene Structure Display Server (GSDS 2.0). The genomic DNA and cDNA sequences of *TtAnns* were determined to clarify the number of exons and introns, as well as the arrangement of each gene. Phylogenetic analysis of the *TtAnn* CDS sequences classified the 15 genes into three distinct groups. Most of the *TtAnns* occurred in pairs (except *TtAnn9*), and the majority of these pairs displayed similar gene structures (except *TtAnn1*/*TtAnn10*, *TtAnn7*/*TtAnn14*, and *TtAnn6*/*TtAnn8*) (Figure 1).

**Figure 2:**
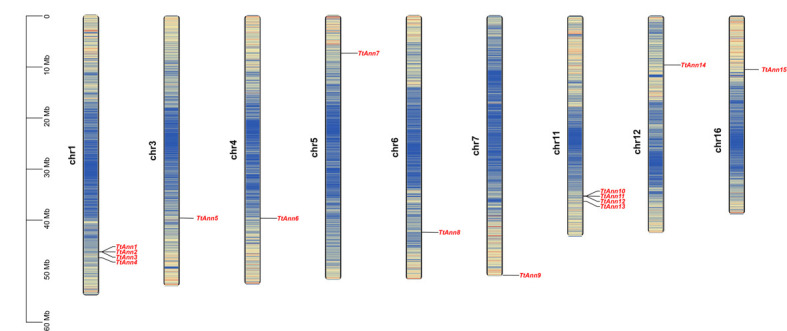
Locations of the 15 *TtAnns* on 16 chromosomes of *T. tetragonoides*.

Chromosomal mapping analysis revealed uneven genomic distribution of the 15 *TtAnns* loci across nine *T. tetragonoides* chromosomes, with no dominant correlation to chromosome length (Figure 2). In total, eight *TtAnns* were mapped on chromosomes 1 (*TtAnn1*, *TtAnn2*, *TtAnn3*, and *TtAnn4*) and 11 (*TtAnn10*, *TtAnn11*, *TtAnn12*, and *TtAnn13*). According to their CDSs’ phylogenetic analysis (Figure 1), *TtAnn1*, *TtAnn2*, *TtAnn3*, *and TtAnn4* are highly homologous with *TtAnn10*, *TtAnn11*, *TtAnn12*, and *TtAnn13*, respectively. Their gene duplication patterns and *ka* and *ks* values also indicated that they were highly homologous gene pairs; and their existence further implied the significance of this gene family for *T. tetragonoides*’ evolution and ecological adaptability. *TtAnn5*, *TtAnn6*, *TtAnn7*, *TtAnn8*, *TtAnn9*, *TtAnn14*, and *TtAnn15* were separately distributed on chromosomes 3, 4, 5, 6, 7, 12, and 16, respectively. The chromosomes 2, 8, 9, 10, 13, 14, and 15 held no *TtAnns*’ loci (Figure 2).

**Table 1:** Nomenclature, properties, and prediction of subcellular localization for the 15 TtAnnexins identified from *Tetragonia tetragonoides*.

Name	Locus	Length (aa) and MW (kDa)	Major Amino Acid (%)	PI	II	AI	GRAVY	TMHs and Topologies *	Annexin Repeats	WoLF_PSORT	Plant-Ploc
TtAnn1	1G0018590.1	323–36.74	L (10.8%), K (8.0%), I (7.7%), S (7.7%)	6.14	38.48	98.98	−0.228	None/inside	3	cyto: 10, nucl: 2, vacu: 1, cytoskel: 1	chloroplast
TtAnn2	1G0018600.1	320–36.43	E (10.9%), L (10.6%), K (9.1%)	6.18	30.42	88.69	−0.37	None/inside	2	cyto: 12, memb: 1, extr: 1	chloroplast
TtAnn3	1G0018610.1	315–36.21	L (11.1%), A (10.5%), E (9.8%)	6.29	40.50	93.59	−0.449	None/inside	2	cyto: 6, nucl: 3, memb: 2, cytoskel: 2, chlo: 1	chloroplast
TtAnn4	1G0019250.1	321–36.60	L (10.3%), K (9.0%), V (8.1%)	9.11	41.35	95.05	−0.262	None/inside	2	chlo: 4, nucl: 3, cyto: 3, mito: 2, memb: 1, pero: 1	chloroplast
TtAnn5	3G0016340.1	316–35.93	L (12.0%), A (9.8%), D (7.9%), E (7.9%), K (7.9%), T (7.9%)	6.04	32.69	92.69	−0.456	None/inside	4	cyto: 9.5, cyto-extr: 5.5, memb: 2, cytoskel: 2	cytoplasm
TtAnn6	4G0016660.1	344–38.90	S (10.2%), L (9.9%), K (8.1%)	9.01	41.99	75.76	−0.556	None/inside	1	nucl: 9.5, nucl-memb: 5.5, cyto: 4	chloroplast
TtAnn7	5G0006300.1	315–35.25	L (10.5%), I (10.2%), A (8.3%)	6.03	34.44	107.14	−0.187	None/inside	3	cytoskel: 10, chlo: 2, cyto: 2	chloroplast
TtAnn8	6G0017790.1	256–28.62	S (10.5%), L (10.2%), A (7.4%), K (7.4%)	9.11	38.89	75.51	−0.546	None/inside	1	nucl: 9.5, cyto-nucl: 7.16667, nucl-memb: 6.33333, cyto: 3.5	nucleus
TtAnn9	7G0020400.1	315–35.58	L (11.4%), A (10.8%), K (7.9%)	9.11	27.7	92.06	−0.302	None/inside	3	chlo: 13, cyto: 1	chloroplast
TtAnn10	11G0018420.1	244–28.41	L (10.7%), E (7.8%), I (7.8%), S (7.8%)	6.85	46.00	95.45	−0.216	None/inside	2	golgi: 5, cyto: 4, chlo: 1, nucl: 1, memb: 1, vacu: 1, extr: 1	chloroplast
TtAnn11	11G0018440.1	320–36.4	E (10.9%), L (10.6%), K (9.1%)	6.25	32.47	87.16	−0.363	None/inside	1	cyto: 11, chlo: 1, memb: 1, extr: 1	chloroplast
TtAnn12	11G0018450.1	315–36.13	L (11.1%), A (10.8%), E (10.2%)	5.99	39.69	92.67	−0.447	None/inside	3	cyto: 8, nucl: 3, memb: 2, cytoskel: 1	chloroplast
TtAnn13	11G0019080.1	321–36.46	L (10.0%), K (8.7%), V (8.1%)	9	40.8	95.67	−0.257	None/inside	2	cyto: 6, chlo: 6, nucl: 2, mito: 1, cytoskel: 1	chloroplast
TtAnn14	12G0005860.1	285–31.94	I (10.5%), L (10.5%), A (8.1%), D (8.1%), K (8.1%)	5.68	37.27	106.46	−0.223	None/inside	3	cytoskel: 11, cyto: 3	cytoplasm
TtAnn15	16G0010050.1	316–35.87	L (12.0%), A (9.8%), D (7.9%), E (7.9%), K (7.9%), T (7.9%)	5.91	33.07	92.69	−0.434	None/inside	4	cyto: 7.5, cyto-extr: 4.83333, cytoskel: 3, extr: 1.33333, chlo: 1, nucl: 1	cytoplasm

* The (https://wolfpsort.hgc.jp/) scores predicted by WoLF PSORT equal to or below 3 were ignored. MW: molecular weight; PI: isoelectric point; II: instability index; AI: aliphatic index; GRAVY: grand average of hydropathicity. The molecular weight and isoelectric points of predicted TtAnns were detected using the ExPASy proteomics server (https://web.expasy.org/protparam/, accessed on 25 November 2025 The contents of disordered amino acids (aa, %) in TtAnns were calculated according to the online program Disordered by Loops/coils definition from DisEMBL 1.5 (Intrinsic Protein Disorder Prediction, http://dis.embl.de/). The TMHMM Server 2.0 program (https://services.healthtech.dtu.dk/services/TMHMM-2.0/, accessed on 25 November 2025) was used to predict the transmembrane helices, and the topologies of TtAnns were also performed with 3D prediction by PHYRE^2^ (http://www.sbg.bio.ic.ac.uk/phyre2/html/page.cgi?id=index, accessed on 25 November 2025). For the subcellular localization prediction, the online program WoLF_PSORT (https://wolfpsort.hgc.jp/, accessed on 25 November 2025) and Plant-PLoc (http://www.csbio.sjtu.edu.cn/bioinf/plant/, accessed on 25 November 2025) were used. WoLF_PSORT predicts the subcellular localization sites of proteins based on both known sorting signal motifs and some correlative sequence features such as amino acid content, and the Plant-Ploc mainly predicts plant proteins with 11 subcellular localizations in cells, including: cell wall, chloroplast, cytoplasm, endoplasmic reticulum, extracellular, mitochondrion, nucleus, peroxisome, plasma membrane, plastid, and vacuole.

**Figure 3:**
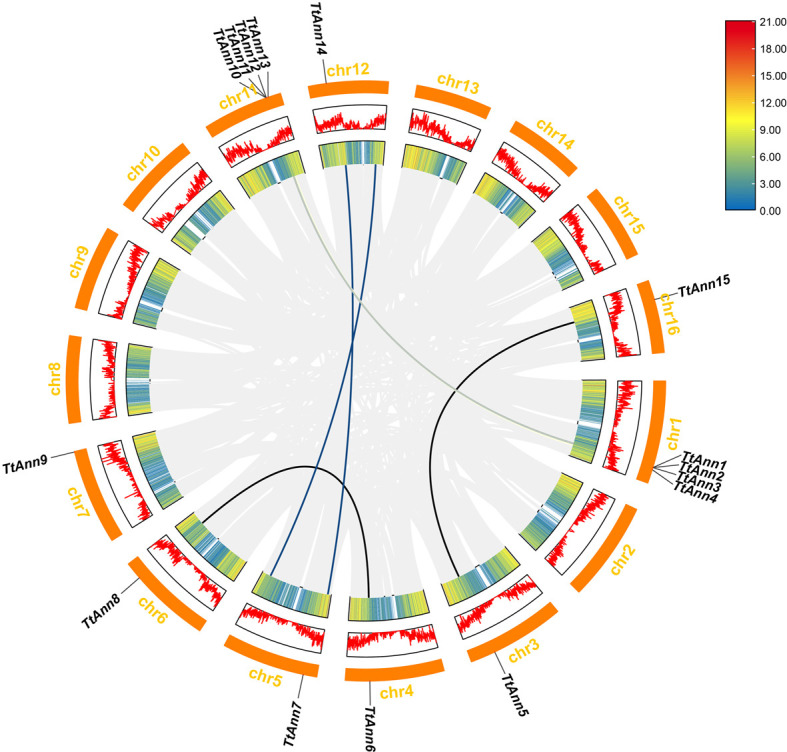
Chromosomal distribution and intragroup covariance analysis of the *TtAnns*. The gray section represents the collinearity of all genes within *T. tetragonoides*; the lines represent the collinearity within the *TtAnn* gene family.

Comparative genomics collinearity analysis was performed with the 15 *TtAnn* members on *T. tetragonoides* chromosomes (Figure 3). The paralog distribution showed strong phylogenetic conservation, mostly occurring in *TtAnn* pairs. Noticeably, the collinearity results further showed the strong phylogenetic conservation between *TtAnn* gene members; and this finding might facilitate functional differentiation and the division of function through the generation of sequence variations. Additionally, the whole-genome duplication and DD events were identified by the presence of duplicated *TtAnn* gene pairs, which were situated on different chromosomes and showed similar patterns (Table S2) as the collinearity analysis results (Figure 3).

### 2.3. Analysis of TtAnns Proteins’ Features and Phylogenetic Evolution Analysis

Although the TtAnn family was identified by the conserved Ann domain (pfam PF00191) search, each TtAnn protein must hold the Ann domain. We summarized the simplified conserved domain search by InterPro and NCBI, and the result showed that each TtAnn had the specific Ann domain with a different length (Figure S1), and their Ann domains contained different Ann repeats (Figure S2). Typically, TtAnn8, TtAnn10, and TtAnn14 had the shortest Ann domain, and their protein lengths were also shorter than those of other TtAnns.

**Figure 4:**
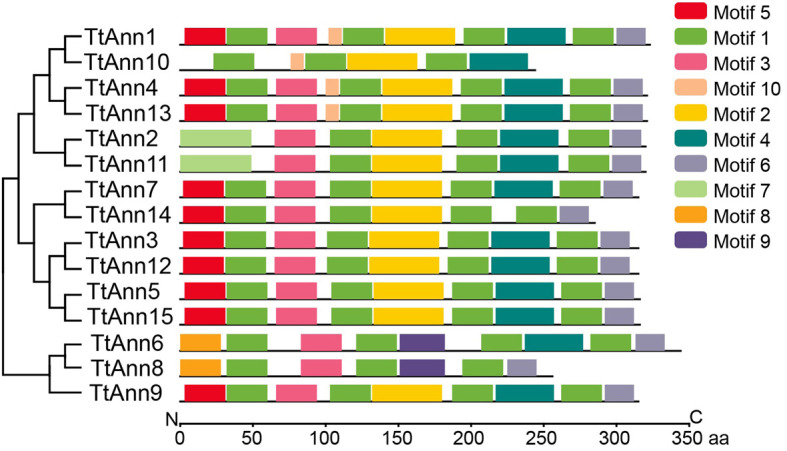
Phylogenetic relationships and motif compositions of the TtAnn proteins. The left part showed the phylogenetic tree constructed with the 15 protein sequences of TtAnns using MEGA X. The right part showed the conserved motifs of each TtAnn analyzed by the MEME web server (http://meme-suite.org/tools/meme). Different motifs are represented by different colored boxes.

**Figure 5:**
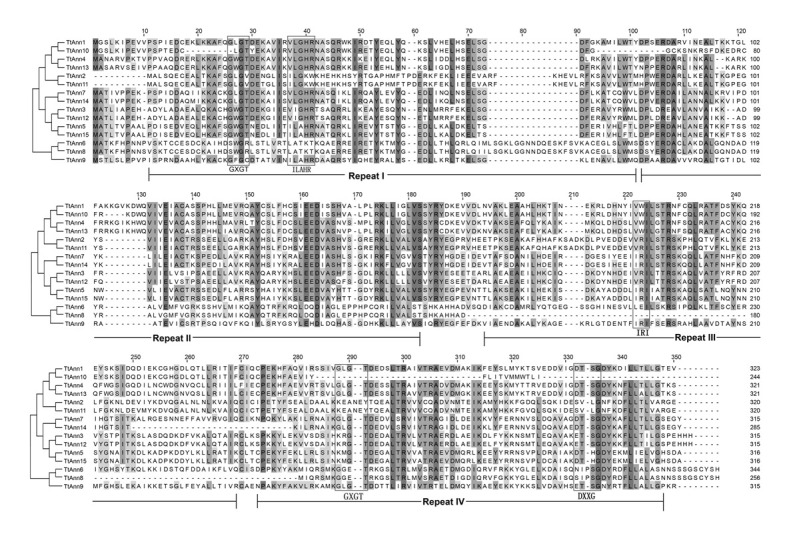
Multiple amino acid sequence alignment of the deduced protein sequences of the 15 TtAnns. Amino acid residues that are conserved are shown in white on a black or gray background. The four repeat regions of all TtAnns were labeled, and the GXGT Ca^2+^-binding sites in repeats I and IV were also marked. The conserved peroxidase residue (ILAHR), the interaction region indicator (IRI) site (binding to filamentous actin), and the DXXG site (binding to GTP), were also labeled.

The conserved regions of the TtAnns were determined with Multiple Em for Motif Elicitation (MEME) analysis, and 10 distinct motifs were identified (Figure 4). Combined with the protein phylogenetic analysis of the TtAnn family, the conserved motifs of evolutionarily similar TtAnn pairs also showed comparable patterns, especially the TtAnn4/TtAnn13, TtAnn2/TtAnn11, TtAnn3/TtAnn12, and TtAnn5/TtAnn15 protein pairs, which presented almost exactly the same conserved motifs. The TtAnn1/TtAnn10, TtAnn7/TtAnn14, and TtAnn6/TtAnn8 protein pairs had differences to varying degrees. Among the 15 TtAnn proteins, only two were the typical Anns, which contained four intact Ann repeats (TtAnn5/TtAnn15) (Figure S2). The Molecular Evolutionary Genetics Analysis (MEGA-X) software was used to further explore the conserved regions of the TtAnn proteins, and the four repeat regions of the TtAnns were labeled (Figure 5). The GXGT Ca^2+^-binding sites in repeats I and IV were also marked. In addition, the conserved peroxidase residue (ILAHR), the interaction region indicator (IRI) site (binding to filamentous actin), and the DXXG site (binding to GTP) were labeled (Figure 5). Overall, the sequence conservation comparative analysis of the TtAnn family showed more considerable differences than the Ann families in the model plant Arabidopsis and its related species (*Schrenkiella parvula* and *Eutrema salsugineum*) [27]. The number of protein members, the distribution of annexin domains, and the evolutionary relationship of the TtAnn family all presented greater diversities than that in Arabidopsis and its two closely related halophytes (Figure 5 and Figure S2).

**Figure 6:**
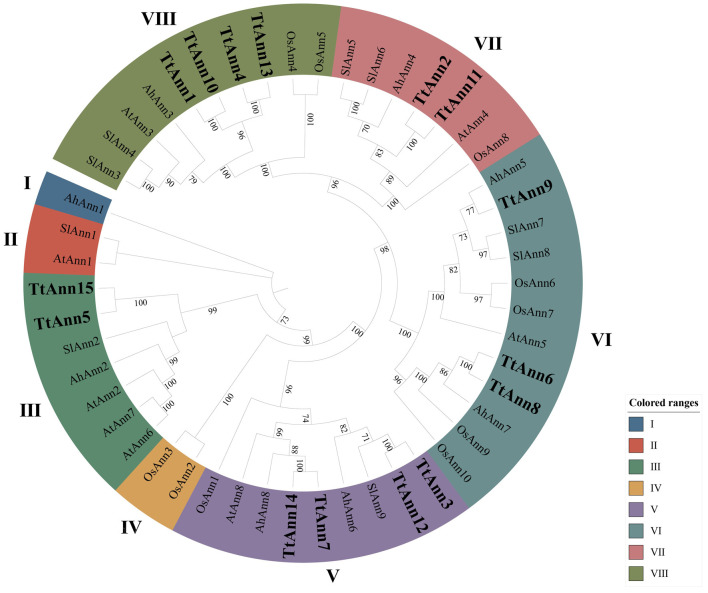
Phylogenetic relationships of the 15 TtAnns from *T. tetragonoides*, 8 AtAnns from *Arabidopsis thaliana*, 10 OsAnns from rice (*Oryza sativa*), 8 AhAnns from peanut (*Arachis hypogaea*), and 9 SlAnns from tomato (*Solanum lycopersicum*). The amino acid sequences of these 50 Anns from five plant species were compared with ClustalW alignment, and the phylogenetic tree was constructed in MEGA X using the maximum likelihood (ML) method, with 1000 bootstrap repetitions. The different branch colors represent different subgroups.

To further explore the underlying evolutionary mechanisms of TtAnns, we selected four representative angiosperm species, namely *A. thaliana* [27], *Oryza sativa* (rice) [11], *Arachis hypogaea* (peanut) [35], and *Solanum lycopersicum* (tomato) [36]. The analysis revealed that the TtAnns exhibited the highest synthetic relationships with the Anns in *A. hypogaea* and *S. lycopersicum* (Figure 6). This evolutionary correlation in the Ann family might be relevant to the relatedness of the species. Overall, a greater number of homologous Ann genes were identified in the genome of *T. tetragonoides* (15), as compared to those found in the genomes of Arabidopsis (8), rice (10), peanut (8), and tomato (9).

### 2.4. Promoter’s Cis-Acting Elements (CEs) of TtAnns

**Figure 7:**
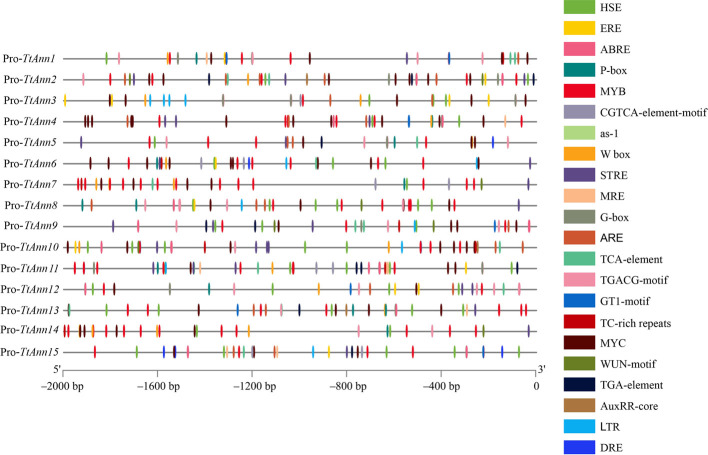
Numbers and distribution of the *cis*-acting elements (CEs) in the 15 *TtAnns*’ promoter regions. Distribution of the sixteen CEs (MYB, ABRE, MYC, ARE, HSE, TGACG-motif, ERE, CGTCA-motif, TC-rich repeat, WUN-motif, TCA element, TATC box, MBS, AuxRR-core, TGA-element, and P-box) were labeled with different color symbols in predicted promoter regions. The scale bar represents 200 bp. The detailed sequences information of CEs was listed in Table S4.

The promoter sequences of *TtAnns* were also investigated to further predict the regulation of gene expression. The genes’ promoter regions could bind to specific transcription factors that have close connections to the CEs. The results showed that 25 different types of CEs were found with promoter-specific patterns (Table S4). Among these, transcription factor (TF)-binding CEs, including MYC, MYB, TGACG-motif (bZIP binding), as-1 (TGA binding), and HSE (HSF binding), were the most abundant, accounting for nearly half of the total. Furthermore, these CEs were distributed in almost all of the *TtAnns*’ promoter regions. Correspondingly, abiotic stress-responsive CEs also presented relatively wide distribution. Unexpectedly, plant growth and development-related CEs, including AuxRR-core, TGA-element, and ERE, were relatively sparse in all promoter regions of *TtAnns* (Figure 7). These results suggested that multiple *cis*-acting elements may be involved in the regulation of *TtAnns* in response to growth, hormone induction, and abiotic stress.

### 2.5. Tissue-Specific Expression Profile of TtAnns

**Figure 8:**
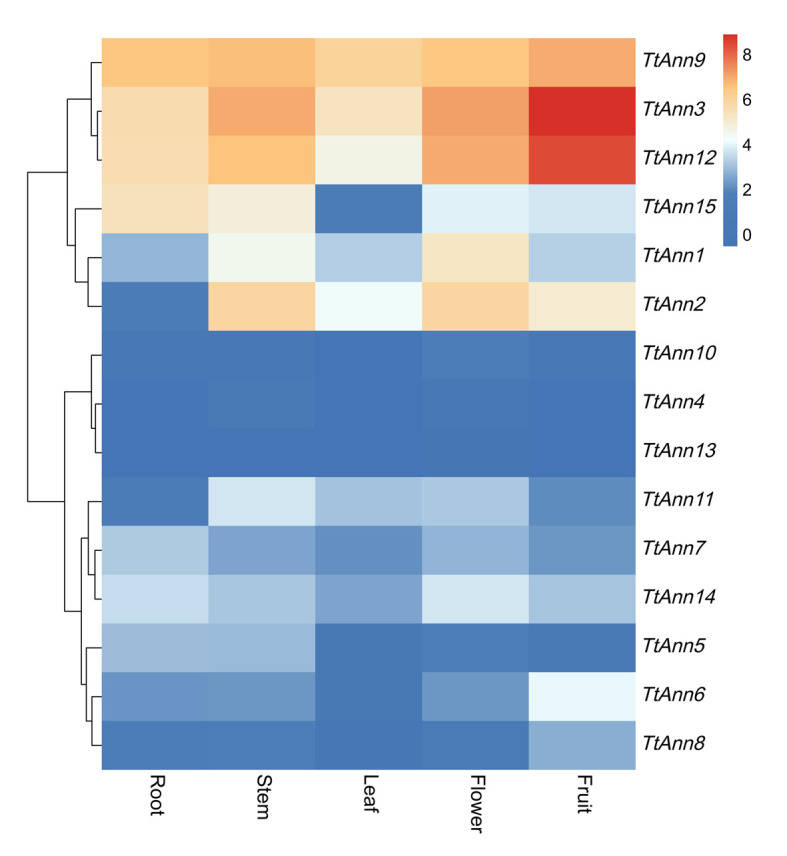
Heatmaps showing the expression levels of the *TtAnns* in the roots, stems, leaves, flower buds, and young fruit of *T. tetragonoides* plants.

The gene expression data from the RNA-seq analysis of the five different organs, namely roots, stems, young leaves, flowers, and young seeds, were used to understand the expression pattern of *TtAnns* in different tissues (Table S5), with the purpose of exploring the putative roles of the *TtAnns* on organ development and branching architecture of *T. tetragonoides*. We found that the *TtAnn3*/*TtAnn12* gene pair showed specific high expression levels in flowers and fruits, while the single *TtAnn9* showed almost uniform stronger expression in all five organs than the other 12 *TtAnns* (Figure 8). Additionally, *TtAnn1* and *TtAnn2* showed obviously higher expression patterns in stems and flowers, indicating that they might be involved in the development of the stems and flowering organs of *T. tetragonoides*. Overall, more than half of the *TtAnn* family members showed a low level of expression in *T. tetragonoides* plants, which might further imply the gene redundancy of the *TtAnn* family.

### 2.6. Expression Analysis of TtAnns in Response to Abiotic Stress in T. tetragonoides

**Figure 9:**
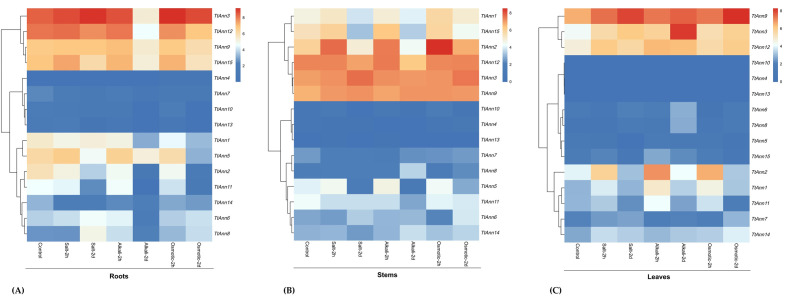
Heatmaps showing the expression levels of the *TtAnns* under salt, alkalinity, and high osmotic treatment in *T. tetragonoides* seedling roots (**A**), stems (**B**), and leaves (**C**). “Control” “−2 h”, and “−2 d” each represent “untreated”, “stress treatment for two hours”, and “stress treatment for two days (2 d, 48 h)”. The heat map was constructed from log2-transformed FPKM (+1) values [log_2_ (FPKM + 1)], and normalized treatments were carried out based on rows. The RNA-seq data of the *TtAnns* were listed in Table S4.

**Figure 10:**
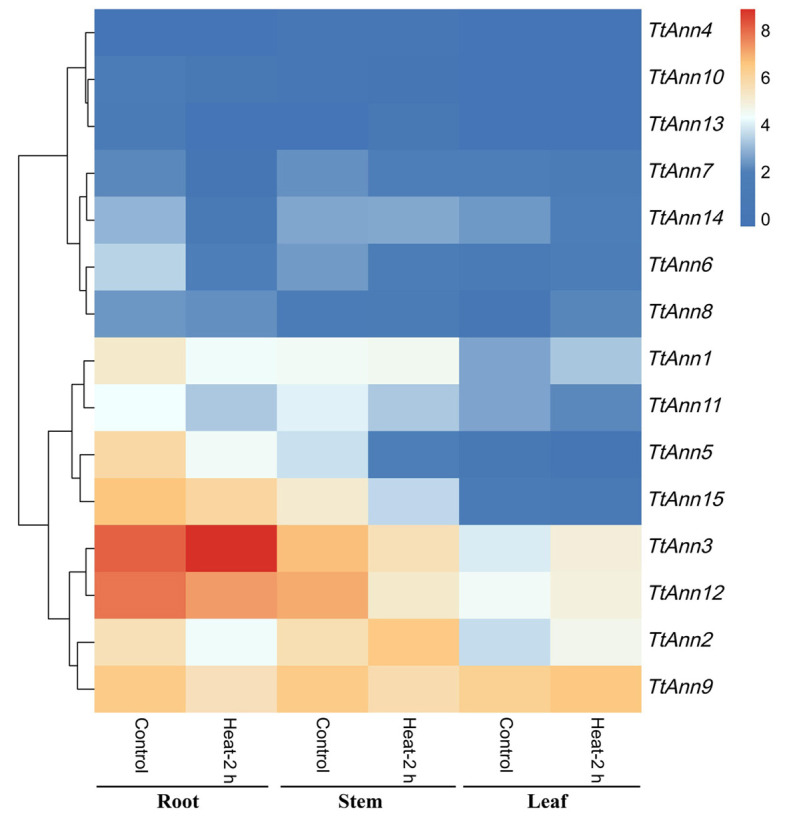
Heatmaps showing the expression levels of the *TtAnns* in *T. tetragonoides* seedlings under heat treatment (45 °C for 2 h). “Control” represents “untreated”. The RNA-seq data of the *TtAnns* were listed in Table S4. The heat map was constructed from log2-transformed FPKM (+1) values [log_2_ (FPKM + 1)], and normalized treatments were carried out based on rows. The RNA-seq data of the *TtAnns* are listed in Table S4.

Promoter analysis (Figure 7) suggested a role for *TtAnns* in *T. tetragonoides* abiotic stress adaptation. Therefore, the expression patterns of the *TtAnns* were further evaluated with RNA-seq (Table S5) and qRT-PCR tests under four different stress challenges: salt (600 mM NaCl), alkali (150 mM NaHCO_3_, pH 8.2), osmotic stress (mimicking drought, 300 mM mannitol), and heat stress (45 °C). As shown in Figure 9A–C, two gene pairs (*TtAnn3*/*TtAnn12* and *TtAnn5*/*TtAnn15*) and the single *TtAnn9* showed obvious changes in regulated expression patterns under the abiotic stress challenges in acute (2 h) and long-term (2 d) treatments, while most of the other *TtAnns* showed mostly low and stable expression levels. Some *TtAnn* genes exhibited significant rapid induction within 2 h of acute stress treatment. These included *TtAnn5* and *TtAnn15* (roots), *TtAnn1*, *TtAnn2*, and *TtAnn5*/*TtAnn15* (stems), and *TtAnn1*, *TtAnn2*/*TtAnn11*, *TtAnn9*, and *TtAnn12* (leaves). Additionally, *TtAnn3* and *TtAnn9* exhibited significant upregulation under long-term stress (2 d), with the notable exception of downregulation during highly toxic alkali stress. Of note, the heat challenge (45 °C, 2 h) was performed on *T. tetragonoides* seedlings, as *T. tetragonoides*, a tropical coastal plant species, has almost inevitably been exposed to extreme heat if it has survived past the seedling stage. *TtAnn3* exhibited marked upregulation in both roots and leaves under heat stress; conversely, it showed slight downregulation in stems. Under heat stress, *TtAnn2* exhibited upregulation in stems, while *TtAnn3* and *TtAnn9* were primarily induced in leaves (Figure 10).

**Figure 11:**
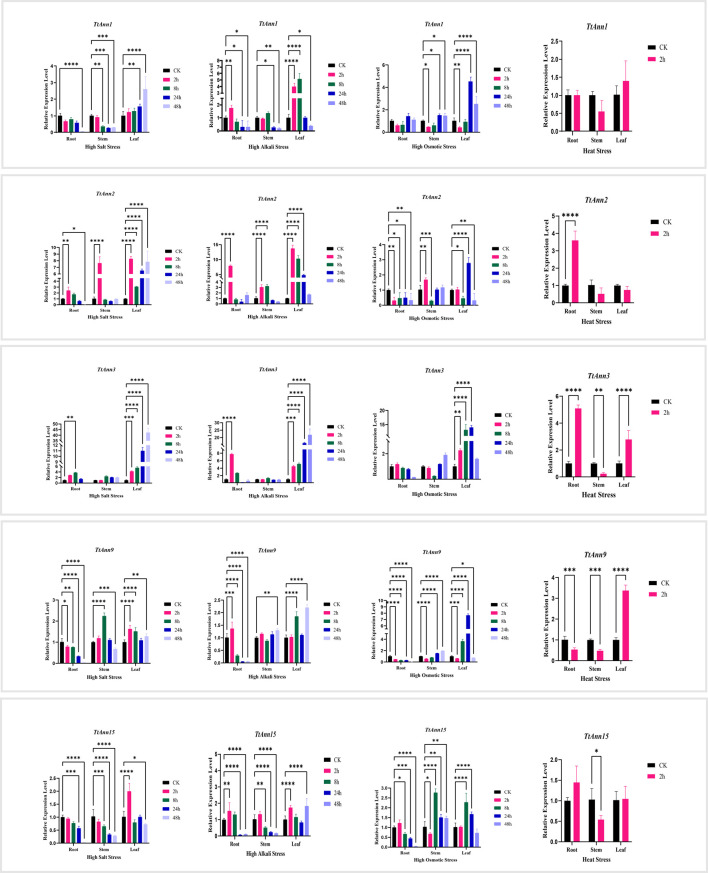
Quantitative RT-PCR detection of the expression levels of the 5 *TtAnns* responding to different stresses (600 mM NaCl for salt, 150 mM NaHCO_3_ for alkali, 300 mM mannitol for artificial drought, and 45 °C for heat) in *T. tetragonoides* seedling plants. Relative expression values were calculated using the 2^−ΔCt^ method with housekeeping gene *TtACT* as reference gene. Bars show mean values ± SD of *n* = 3–4 technical replicates. Asterisks indicate significant differences: * *p* < 0.05, ** *p *< 0.01, *** *p *< 0.001, and **** *p *< 0.0001.

Further qRT-PCR analysis was performed for five specific *TtAnns*, including *TtAnn1*, *TtAnn2*, *TtAnn3*, *TtAnn9*, and *TtAnn15* (Figure 11), as they showed more obvious changes in regulated expression patterns than the other 10 *TtAnns* and are considered key genes for multiple abiotic stress responses. Our results demonstrated that the expression levels of these five *TtAnns* were significantly altered under abiotic stress (Figure 9, Figure 10 and Figure 11), providing further evidence that these genes play critical roles in modulating the environmental adaptability of *T. tetragonoides*.

### 2.7. Functional Characterization of TtAnns in Yeast

**Figure 12:**
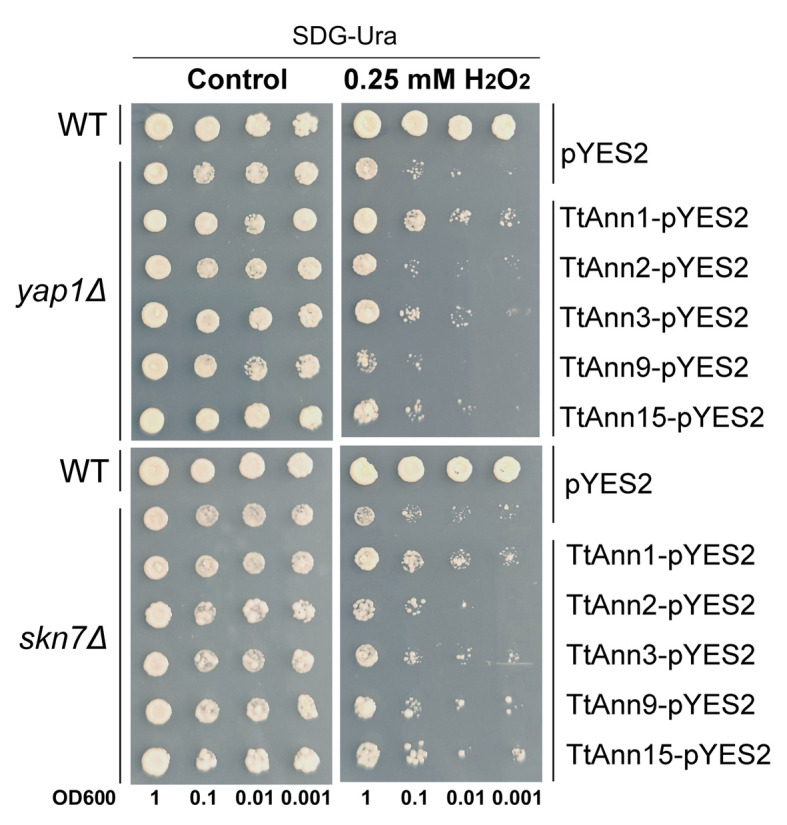
Functional identification related to hydrogen peroxide (H_2_O_2_) tolerance of six *TtAnns* in yeast using heterologous expression assays. The yeast wild-type (WT) and two H_2_O_2_-sensitive mutant strains, *skn7∆* and *yap1∆*, were transformed with the empty vector pYES2 or six recombinant vectors, namely TtAnn1-pYES2, TtAnn2-pYES2, TtAnn3-pYES2, TtAnn9-pYES2, and TtAnn15-pYES2.

**Figure 13:**
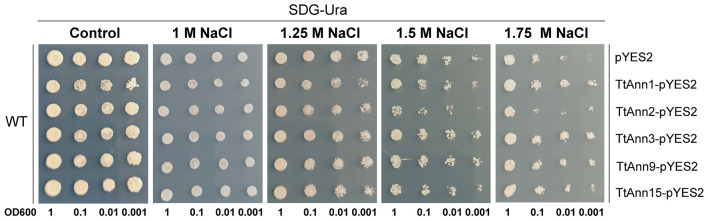
The salt (NaCl) tolerance confirmations of the 5 *TtAnns*’ heteroexpression in wild type (WT) yeast.

**Figure 14:**
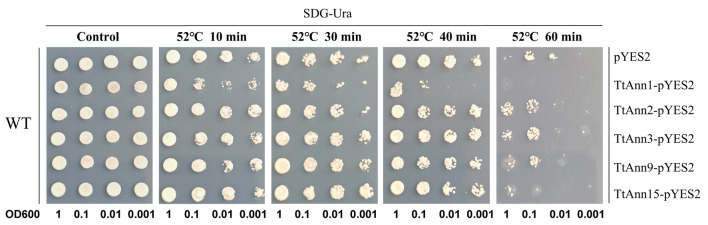
Functional identification of 5 *TtAnns*’ function related to heat challenges (52 °C for 10, 30, 40, and 60 min, WT yeast strain without heat stress as control).

To further validate the results of the transcriptional profiling of the *TtAnns*’ biological functions involved in multiple abiotic stress responses, we performed gene cloning and functional verification with a yeast system. Yeast lacks endogenous *Ann* genes [37], previous reports have demonstrated that heterologous expression of Ann genes in yeast could affect the stress tolerance of different yeast strains [38,39]. The analysis of the physicochemical properties of TtAnns by different programs indicated that these proteins might play roles in cytoplasm or chloroplast (Table 1). Here, we predicted TtAnns acting as cytoplasmic soluble proteins and playing stress-resistance intracellular functions. There is no doubt that the expression of *TtAnns* with the yeast system would help in understanding the biological roles involved in multiple stress tolerances mediated by specific TtAnns. Combined with the results of transcriptome analysis and evolution of the *TtAnn* family, we suspect that the genes with a relatively high-level expression or more obvious expression alterations under stress challenges might undertake more important functions. Therefore, the full-length CDSs for the five candidate *TtAnns*, *TtAnn1*, *TtAnn2*, *TtAnn3*, *TtAnn9*, and *TtAnn15*, were inserted into the yeast expression vector pYES2 to transform different yeast strains and gain further insight into the functions of *TtAnns* in the single-cell model eukaryote yeast. We evaluated the hydrogen peroxide (H_2_O_2_) tolerance of the yeast transformants expression the five *TtAnns*, as Anns have been reported to possess peroxidase activity [10,11]. As we can see from Figure 12, when using *yap1Δ* and *skn7Δ* yeast strains for H_2_O_2_ tolerance tests, only *TtAnn1*, *TtAnn3*, and *TtAnn15* presented slightly stronger antioxidant capacities than the control (empty vector pYES2). Additionally, *TtAnn1*, *TtAnn3*, *TtAnn9*, and *TtAnn15* could slightly elevate the tolerance of wild type yeast strain (WT, BY4741) to salt (1.75 M NaCl) (Figure 13). Unexpectedly, *TtAnn2* conferred only marginal increases in tolerance to H_2_O_2_ and NaCl (Figure 12 and Figure 13), which might be related to the specific sub-cellular localization or protein structure of *TtAnn2*. Notably, *TtAnn1* significantly enhanced the heat tolerance (52 °C) of the WT yeast, potentially due to the formation of ion channels in the plasma membrane. The other four *TtAnns*, namely *TtAnn2*, *TtAnn3*, *TtAnn9*, and *TtAnn15*, each conferred a marginal increase in heat tolerance (Figure 14). This effect may be related to the protective effects of their peroxidase activity or actin-binding chaperone behavior. Further in-depth functional analysis of these TtAnns using plant transgenic assays is required to confirm these findings.

## 3. Discussion

Anns are widely distributed proteins both at the species level [2,3] and at the cell or tissue levels [8,9]. Consequently, their regulatory roles in plant growth, development, and stress responses have been extensively investigated. The Ann family has been identified, and some members have been functionally analyzed in various plant species, including model plants, wild plants, and crops [2,7]. The integrated analysis of genome, proteome, transcriptome, and metabolome data has significantly accelerated the functional characterization of this gene family. Consequently, the plant Ann family has emerged as a prominent focus in plant molecular biology [33,40]. In this study, the *TtAnn* gene family was comprehensively characterized. Given the limited research on *Ann* genes from plants in special habitats, especially from halophytes, more in-depth studies are essential to inform future crop improvement strategies. In this study, 15 *TtAnn* genes were identified using genomic and transcriptomic data of *T. tetragonoides*. This halophyte vegetable is native to tropical and subtropical coastal regions, which are characterized by high salinity, alkalinity, severe drought, extreme temperatures, and high-calcium environments.

As previously reported, plant Anns have some conserved motifs and residues, which are important for their activity. These include Ca^2+^ binding sites, conserved tryptophan for membrane binding, His40 for peroxidase activity, ATP/GTP binding motif, and F-acting binding motif [27]. Furthermore, the presence of a conserved Ann domain at the C-terminal is the typical characteristic of this protein family. In this study, 15 *TtAnn* genes were systematically analyzed based on gene evolution, protein profiles, transcription analysis, and initial function identification. Previous research about the annexin families in Arabidopsis and its closely related halophytes *S. parvula* and *E. salsugineum* further implied that the gene duplication events and transcriptional regulation for *annexins* might be variable regulatory mechanisms that contribute to salt tolerance and their natural ecological adaptability for halophytes [27]. Because *T. tetragonoides* is remarkably tolerant of its harsh native environment, researchers believe that its Ann proteins are a key factor in its ecological adaptability.

Plant Anns, belonging to Ca^2+^-dependent lipid-binding proteins, constitute a multifunctional and multigene family. The Ann family members have conservative functional domains, often containing four homologous repeated domains [6,7]. The motifs in the TtAnns’ domains also presented a strong level of similarity across the members (Figure 4 and Figure S1). Similarly, the structures of the TtAnns were conserved, and the four repeats in the Ann domains were relatively conserved in amino acid sequences (Figure 5 and Figure S2). In fact, based on the biochemical function of Anns, this group of proteins also constitutes specific membrane stabilization re-sealing [1] or acts as intracellular membrane channels [2]. Here, we highlighted the adaptive evolution and sequence conservation of the TtAnn family in *T. tetragonoides*, and these comprehensive analyses may help our understanding of possible roles involved in stress responses mediated by specific plant Anns.

The genes’ expression was precisely regulated by a series of transcription complexes, which were manipulated by some key transcription factors (TFs). The *cis*-acting elements, CEs, in promoter regions are quite crucial for genes’ expression and the subsequent performance of their biological functions. Given its original habitat, *T. tetragonoides* frequently encounters high salinity, alkalinity, drought, heat, and intense sunlight during its growth. Consequently, the regulated expression of *TtAnns* under their respective promoters is critical for the survival and adaptation of this species. In this study, we focused on some CEs in the promoters of *TtAnns*, specifically ABRE, HSE, MYC, and MYB. The results indicated that each *TtAnn* was precisely regulated by multiple TFs (Figure 7 and Table S4). These findings provide a preliminary transcriptional framework for further functional research on plant *Anns*.

Additionally, the gene expression of the *TtAnns* was also analyzed in various tissues under different stages of growth and development. In Arabidopsis, the expression profiling of the *AtAnn* family across various developmental stages and environmental stresses highlights its essential roles in growth and stress adaptation [13]. Only fewer than half of the *TtAnn* members—specifically *TtAnn1*, *TtAnn2*, *TtAnn3*/*TtAnn12*, *TtAnn9*, and *TtAnn15*—exhibited strong expression levels in all five *T. tetragonoides* tissues, showing distinct development-dependent patterns (Figure 8). Comparatively, the *Ann* family frequently exhibited low basal expression across various plant species, with only a few members showing predominant expression levels; similar patterns have been reported in radish [24], rye [25], and chickpea [28]. Conspicuously, *TtAnn3*/*TtAnn12* were expressed much higher in young seeds of *T. tetragonoides* (Figure 8), indicating their potential functional roles in seed development and maturation. Also, *TtAnn5*/*TtAnn15* presented relatively high expression levels in roots of *T. tetragonoides* seedlings (Figure 9 and Figure 10), which reflected this gene pair playing more important roles for the development of roots and more direct gene expression regulation to abiotic stress response. However, the *TtAnn* members did not exhibit uniform tissue-specific expression patterns, and further functional studies are required to determine whether these expression profiles correspond to specific developmental functions.

Plant Anns have been implicated in plant responses to diverse environmental stresses and pathogen infections [6,7,8,9,10]. It has been proposed that Ann proteins mitigate the damage caused by salinity, drought, and temperature extremes through their specific biochemical activities, including peroxidase, ATPase/GTPase activity, as well as Ca^2+^ channel activity [16,41]. Soil salinization is a major constraint on plant growth and development worldwide. In cotton (*Gossypium* spp.), the Ann protein GhANN8b has been shown to interact with phosphatase GhDsPTP3a, thereby regulating cotton’s tolerance to salt stress [42]. Overexpression of the wild tomato (*Solanum pennellii*) Ann gene *AnnSp2* in transgenic plants enhances salinity and drought tolerance [43]. Additionally, expression of the *Ann* gene families was affected by these abiotic stress challenges in three Brassicaceae species [5], pepper (*Capsicum annuum*) [23], rye (*Secale cereale*) [25], crape myrtle (*Lagerstroemia indica*) [29], and grape (*Vitis vinifera*) [31]. In this study, the expression patterns of the *TtAnn* family were detected via RNA-seq in *T. tetragonoides* seedlings subjected to high salinity (600 mM NaCl), high alkalinity (150 mM NaHCO_3_, pH8.2), high osmotic stress (mimicking drought, 300 mM mannitol), and heat challenge (45 °C). Overall, acute abiotic stress (2 h and 8 h) significantly induced the expression of several *TtAnn* members. In contrast, long-term stress (2 d) generally resulted in the downregulation of most genes in the family (Figure 9, Figure 10 and Figure 11). This downregulation may be attributed to the accumulation of physiological damage in *T. tetragonoides* seedlings under prolonged stress.

The biological functions of several *TtAnns* were preliminarily verified with the yeast expression system. Because yeast strains lack endogenous *Ann* homologs [37,39], their heterologous expression provided a clean genetic background to investigate the specific roles of TtAnn proteins in abiotic stress tolerance. Also in plant transgenic systems, the over-expression of *annexin* genes further verified the biological functions of specific annexins. For example, Arabidopsis annexins *AnnAt1*, *AnnAt4*, and *AnnAt8* have been proved to be involved in salt, drought, and heat tolerance [14,15,16,17,44]. The over-expression of wild tomato *AnnSp2* in cultivated tomato could increase the plants’ tolerance to drought and salt challenges, and the various physiological parameters measure indicated that the accumulation of AnnSp2 might induce stomatal closure and reduce water loss, as well as elimination of cellular ROS [43]. Constitutive expression of *Brassica juncea* annexin *AnnBj2* confers salt tolerance in mustard transgenic plants, probably by affecting the ABA-mediated signal pathway [45]. Also, the over-expression of rice *OsANN4* [46] and *OsANN9* [47] could affect the ABA sensitivity and drought tolerance of transgenic rice plants, probably by modulating ROS scavenging. The grape *VvANN8* [31] and cassava *MeAnn2* [48] could improve the transgenic Arabidopsis plants’ salt tolerance, probably by mediating Ca^2+^ signaling or modulating the levels of MDA, H_2_O_2_, and O^2−^, thereby participating in ROS elimination under salt stress. The radish *RsANN1a* [24] and peanut *AhANN6* [49] promoted heat resistance in transgenic Arabidopsis plants by maintaining ROS detoxification. In this study, given the potential peroxidase activities of TtAnns, we evaluated the H_2_O_2_ tolerance of yeast transformants under galactose-induced expression. As we can see from Figure 12, only some *TtAnns* (*TtAnn1*, *TtAnn3*, and *TtAnn15*) conferred a marginal increase in H_2_O_2_ tolerance of the yeast strains *yap1Δ* and *skn7Δ*. These results are consistent with the effects observed for *Brassica juncea* annexin-3 [38] and *Endocarpon pusillum* EpANN [39]. Accumulating evidence suggests that plant Anns have been involved in multiple abiotic stress responses [15,41] and that the level of cellular reactive oxygen species (ROS) accumulation following stresses in vivo is the basic characteristic of stress response or tolerance. Plant *Anns* respond to abiotic stresses, with similar multiple expression patterns across key plant lineages. In terms of molecular function, heterologous induced expression of *TtAnns* in yeast also enhanced the stress tolerance of different strains (Figure 13 and Figure 14), which may be clear evidence that the *TtAnns* play important protective roles in protecting yeast cells from oxidative damage by scavenging ROS or mitigating other abiotic stress-induced effects.

## 4. Materials & Methods

### 4.1. Plant Materials and Stress Treatments

*Tetragonia tetragonoides* seedlings were grown in a greenhouse at 22–26 °C with a 16 h light/8 h dark photoperiod. The relative humidity of the growth substrate (mixture of vermiculite and peat moss) was controlled at approximately 60%. To investigate the organ-specific expression pattern of *TtAnns*, different organ samples including roots, stems, leaves, flower buds, and young seeds were collected from 3-month old plants. Two-month-old seedlings in similar growth conditions were used for abiotic treatments.

To simulate the native habitats of *T. tetragonoides* plants, the seedlings were transferred to different challenge environments to complete the stress treatments as in our previous report [50]. In brief, for high salt stress, high alkalinity stress, and high osmotic stress (mimicking drought), the *T. tetragonoides* seedlings were removed from their vermiculite pots, carefully washed with water to remove matrix from the roots, and transferred into 600 mM NaCl (similar with the salt concentration of seawater, about 3.5% NaCl), 150 mM NaHCO_3_ (pH 8.2), 300 mM mannitol solutions, respectively. For heat stress, *T. tetragonoides* seedlings in growth pots were moved to a 45 °C illumination incubator for 2 h. The seedlings grown at 22–26 °C were set as control. The roots, stems, and young leaves from the *T. tetragonoides* seedling were collected 2 and 48 h after stress treatments, and all samples were immediately frozen in liquid nitrogen after picking and stored at −80 °C for follow-up experiments.

### 4.2. Genome-Wide Identification of Annexin Genes in T. tetragonoides

The *T. tetragonoides* genome was sequenced and submitted to the NCBI database (NCBI accession number: JBBMRK000000000.1). All *T. tetragonoides* proteins were identified with InterProscan (https://www.ebi.ac.uk/interpro/search/sequence/ (accessed on 22 September 2025)), and the conserved domains and motifs (e < 1 × 10^−5^) were assessed. To characterize the *annexin* family of the *T. tetragonoides* genome, the conserved annexin domain (pfam No. PF00191, or InterPro No. IPR018502) was set as a model, and the protein sequences containing this domain were screened using HMM3.0 software. The domains were also confirmed using the NCBI CDD program (https://www.ncbi.nlm.nih.gov/cdd/, accessed on 29 October 2025). The conserved motifs of all TtAnn proteins were also identified with MEME web server (http://meme-suite.org/index.html, accessed on 29 October 2025). The *T. tetragonoides* proteins with the annexin domain and corresponding genes were eventually identified as the *TtAnnexin* (*TtAnn*) family. The sequences’ information of 15 *TtAnns*, including genes, coding sequences of cDNA (CDS), coding proteins, and predicted promoters, was listed in the Table S1.

### 4.3. Biochemical Features of the TtAnn Proteins and Chromosomal Location, Ka/Ks Calculation, and Phylogenetic Analysis of TtAnns

The molecular weight (Mw), isoelectric point (pI), and grand average of hydropathicity (GRAVY) values of TtAnn proteins were predicted using the online tools (https://web.expasy.org/protparam/, accessed on 25 November 2025). The subcellular localization was predicted with WoLF_PSORT (https://wolfpsort.hgc.jp/, accessed on 25 November 2025), and Plant-mPLoc (http://www.csbio.sjtu.edu.cn/bioinf/plant-multi/, accessed on 25 November 2025) programs.

The loci of *TtAnns* were obtained from the *T. tetragonoides* genome annotation data. Online tool MG2C v2.1 (http://mg2c.iask.in/mg2c_v2.1/, accessed on 25 November 2025) was applied to map the chromosome locations for *TtAnns*. The nonsynonymous substitutions per nonsynonymous site (*Ka*), the number of synonymous substitutions per synonymous site (*Ks*), and the probability (*p*-value) of Fisher’s exact test of neutrality were calculated to explore the selective pressures on the duplication of *TtAnns* based on all nucleotide sequences using the Nei-Gojobori model with 1000 bootstrap replicates. A *Ka*/*Ks* ratio < 1 indicates purifying selection, a *Ka*/*Ks* ratio = 1 indicates neutral selection, and a *Ka*/*Ks* ratio>1 indicates positive selection [5]. Gene whole genome duplication (WGD) and dispersed duplication (repetitive genes that are neither adjacent nor collinear) (DD) events of the *TtAnn* family members were analyzed using MCScanX software (X version) (http://chibba.pgml.uga.edu/mcscan2/, accessed on 25 November 2025). The duplication patterns of *TtAnns* were summarized in Table S2, and the *Ka*/*Ks* values were listed in the Table S3.

The protein sequences of annexins of *T. tetragonoides* (15 members), Arabidopsis (8 members) [2], rice (10 members) [40], peanut (8 members) [35], and tomato (9 members) [36] were retrieved accordingly. The TtAnns and the above species’ annexins were used to construct phylogenetic trees using MEGA X according to the maximum likelihood (ML) method [51]. The protein sequences of the above plants’ annexins were listed in Table S1. The phylogenetic tree was constructed and bootstrap testing was performed with 1000 iterations.

### 4.4. Determination of the Gene (Including Promoter) and Protein Structures of the T. tetragonoides Annexins

The *TtAnn* genomic sequences and coding sequences (CDSs) extracted from the *T. tetragonoides* genome sequencing file were compared in the Gene Structure Display Server 2.0 (http://gsds.cbi.pku.edu.cn) to determine the exon/intron organization of *TtAnns*.

Putative *TtAnn* promoter sequences (2000 bp upstream of ATG) were retrieved from the *T. tetragonoides* genome database (Table S1) and uploaded to the PlantCARE database (http://bioinformatics.psb.ugent.be/webtools/plantcare/html/, accessed on 25 November 2025) for *cis*-acting element (CE) analysis. The CEs were classified as either hormone-specific (ERE, P-box, AuxRR-core, TCA-element, TGA-element, as-1, JERE, and ABRE), plant growth and development-related elements (AuxRR-core, TGA-element, ERE), and abiotic stress-responsive (MYC, MYB, MBS, STRE, ARE, LTR, TC-rich repeats, WUN-motif, DRE, MRE, TGACG-motif, CGTGA-motif, LTRE, ABRE, and HSEs). The CEs were summarized in Table S4. *TtAnn* promoters were visualized using TBtools-II [52].

The protein sequence alignment of TtAnns were performed with online program Jalview (https://www.jalview.org/, accessed on 25 November 2025), and their annexin repeats (I to IV) were labeled manually according to previous report [23]. The NCBI Conserved Domain Search Service (CD Search) (https://www.ncbi.nlm.nih.gov/Structure/cdd/wrpsb.cgi, accessed on 25 November 2025) was also used to confirm the annexin repeats of TtAnns. The conserved protein motifs of all TtAnns were detected by using the online tool Multiple Em for Motif Elicitation (MEME) (http://meme-suite.org/) program with the number of different motifs set to 10. The conserved domains of TtAnns were also analyzed with NCBI Conserved Domain Database (CDD) (https://www.ncbi.nlm.nih.gov/cdd, accessed on 25 November 2025) and InterPro (https://www.ebi.ac.uk/interpro/, accessed on 25 November 2025) search.

### 4.5. RNA-Seq of TtAnns in Different T. tetragonoides Tissues Under Different Stress Treatments

A transcriptome database was constructed for *T. tetragonoides* using Illumina HiSeq X sequencing technology. The quality of the RNA-seq datasets created from five organs (roots, stems, young leaves, flowers, and young seeds collected from *T. tetragonoides* growing in the SCBG) was examined using FastQC (v0.12.1) (http://www.bioinformatics.babraham.ac.uk/projects/fastqc/, accessed on 20 November 2023), which produced 40 Gb of clean reads. Clean reads were mapped to the *T. tetragonoides* reference genome using Tophat v.2.0.10 (http://tophat.cbcb.umd.edu/, accessed on 20 November 2023). The gene expression levels were calculated as fragments per kilobase of transcript per million mapped reads (FPKM) according to the length of the gene and the read counts mapped to the gene: FPKM = total exon fragments/[mapped reads (millions) × exon length (kb)]. The expression levels [log_2_ (FPKM + 1)] of *TtAnns* were visualized as heatmaps using TBtools. The FPKM values for all samples are listed in Table S5.

After different stress treatments, the *T. tetragonoides* seedlings were split into three parts, including roots, stems, and leaves. Then these tissues were used to isolate total RNAs for further RNA-seq or qRT-PCR analysis. Tissue-specific expression profiles of *TtAnns* with or without stress challenges were analyzed using RNA-seq in the roots, stems, leaves, flower buds, and young fruit of *T. tetragonoides* plants. This assay were commissioned by OECloud (https://cloud.oebiotech.com, accessed on 20 November 2023 Oebiotech Co., Shanghai, China) and related data analyses were carried out accordingly.

### 4.6. Expression Pattern Analysis Using Quantitative Reverse Transcription (qRT)-PCR

Specifically, five *TtAnns*’ expression patterns were further confirmed with qRT-PCR. Total RNA was extracted from *T. tetragonoides* seedlings using the EasyPure^®^ Plant RNA Kit (TransGen Biotech, Beijing, China). The RNAs were quantified using a NanoDrop1000 (NanoDrop Technologies, Inc., Wilmington, DE, USA) spectrophotometer, and their integrity was checked on a 0.8% agarose gel. After that, the cDNAs were synthesized using the cDNA Synthesis SuperMix kit (TransGen Biotech, Beijing, China) following the manufacturer’s instructions. The expression analysis of five *TtAnns*, including *TtAnn1*, *TtAnn2*, *TtAnn3*, *TtAnn9*, and *TtAnn15*, was detected by qRT-PCR with the LightCycler480 system (Roche, Basel, Switzerland) and the TransStart Tip Green qPCR SuperMix (TransGen Biotech, Beijing, China). The genes’ expression data obtained via qRT-PCR were normalized to the reference gene *TtACT*’s expression (NCBI accession No.: MH33308). The primers used for qRT-PCR (TtACTRTF/TtACTRTR as the reference gene and other gene-specific primer pairs) were listed in Table S1.

### 4.7. Functional Identification with Yeast Expression System

Five *TtAnns* (*TtAnn1*, *TtAnn2*, *TtAnn3*, *TtAnn9*, and *TtAnn15*) were PCR-cloned with a cDNA sample of *T. tetragonoides* seedlings as templates. In brief, the open reading frames (ORFs) of candidate *TtAnns* were PCR-amplified with gene-specific primer pairs (Table S1). The PCR fragments were purified and then inserted into the *Bam*H I and *Eco*R I sites of the yeast expression vector pYES2 to yield recombinant plasmids of TtAnns-pYES2. Then, these constructed vectors were sequenced before being transformed into yeast cells to prove these sequences to be correct. Different yeast strains were used in this study, including H_2_O_2_-sensitive mutant strains *skn7Δ* (MATa; *his3Δ1*; *leu2Δ0*; *met15Δ0*; *ura3Δ0*; YHR206w::kanMX4; accession number: Y02900), *yap1Δ* (MATa; *his3Δ1*; *leu2Δ0*; *met15Δ0*; *ura3Δ0*; YML007w::kanMX4; accession number: Y00569) and their corresponding wild type (WT) strain BY4741 (MATa; *his3Δ1*; *leu2Δ0*; *met15Δ0*; *ura3Δ0*; accession number: Y00000) were obtained from Euroscarf (http://www.euroscarf.de/index.php?name=News, accessed on 1 April 2017). The standard polyethylene glycol (PEG)–lithium acetate-based transformation procedure was used for yeast plasmid transformation with amino acid defect screening on SDG (-Ura) medium from Chundu Bio. (Wuhan, China. https://chundubio.biomart.cn). The yeast spot assays for H_2_O_2_, NaCl, and heat tolerance were performed as previously described [53].

### 4.8. Statistical Analysis

All experiments in this study were repeated independently three times, and the results are shown as the mean ± standard deviation (SD) (*n* ≥ 3). Pairwise differences between means were analyzed using a Student’s *t*-test in Microsoft Excel 2021.

## 5. Conclusions

In conclusion, the *TtAnn* gene family, comprising 15 novel members, was systematically identified in the present study. Sequence analysis revealed that these TtAnns possess Ann-specific domains, while exhibiting significant sequence divergence within their N-terminal region. Furthermore, phylogenetic tree analysis showed that the *TtAnns* together with Anns from *Arabidopsis*, peanut, tomato, and rice were clustered into five main groups. Chromosomal localization showed that these 15 *TtAnns* were distributed on nine chromosomes of *T. tetragonoides*. The presence of multiple promoter CEs and the tissue-specific expression patterns of *TtAnns* suggest that this family utilizes divergent regulatory mechanisms to mediate resistance to various abiotic stresses. Overexpression of five *TtAnns* in yeast demonstrated their potential roles in conferring salt and heat stress tolerance in *T. tetragonoides*.

## Data Availability

The original contributions presented in this study are included in the article/Supplementary Material. Further inquiries can be directed to the corresponding author.

## Supplementary Materials

The following supporting information can be downloaded at: https://www.mdpi.com/article/10.3390/plants15142218/s1, Figure S1. The annexin domains containing motifs of TtAnns identified by NCBI. Figure S2. The pfam domain diagrams of 15 TtAnns identified on the InterPro website (http://pfam.xfam.org/). Table S1. The obtained *TtAnns*’ nucleotide, protein sequences information, and primer sequences in this study. Table S2. The *TtAnns*’ gene locus and duplication pattern. Table S3. *TtAnn* family Ka and Ks values. Table S4. The categories of *cis*-acting elements (CEs) identified in the *TtAnns*’ promoter regions. Table S5. FPKM values of *TtAnns* for the RNA-seq assay of *Tetragonia tetragonoides* tissues.
