# Supplementary material for: Comprehensive Analysis of Annexin Family from Tetragonia tetragonoides and Its Initial Functions in Abiotic Stress Responses
Source: Plants (Basel). 2026 Jul 21;15(14):2218. doi: 10.3390/plants15142218 (PMC13415601; doi:10.3390/plants15142218)

Supplementary materials

Comprehensive Analysis of Annexin Family from *Tetragonia tetragonoides* and Its Initial Functions in Abiotic Stress Responses

Lihua Chen [1, 2], Fuying Xie [1, 2], Shuguang Jian [1, 3], Zhengfeng Wang [1, 3], Tingyao Li [4], Mei Zhang [1, 3,*]

1 Center of Economic Botany, Core Botanical Gardens; Guangdong Provincial Key Laboratory of Applied Botany, South China Botanical Garden, Chinese Academy of Sciences, Guangzhou, 510650, China.

2 University of the Chinese Academy of Sciences, Beijing 100039, China.

3 Southern Marine Science and Engineering Guangdong Laboratory (Guangzhou), Guangzhou, China

4 Vegetable Research Institute, Guangdong Academy of Agricultural Sciences, Guangzhou, Guangdong 510640, China.

*Correspondence and requests for materials should be addressed to Mei Zhang (zhangmei@scbg.ac.cn).

Figure S1 The annexin domains containing motifs of TtAnns identified by NCBI.

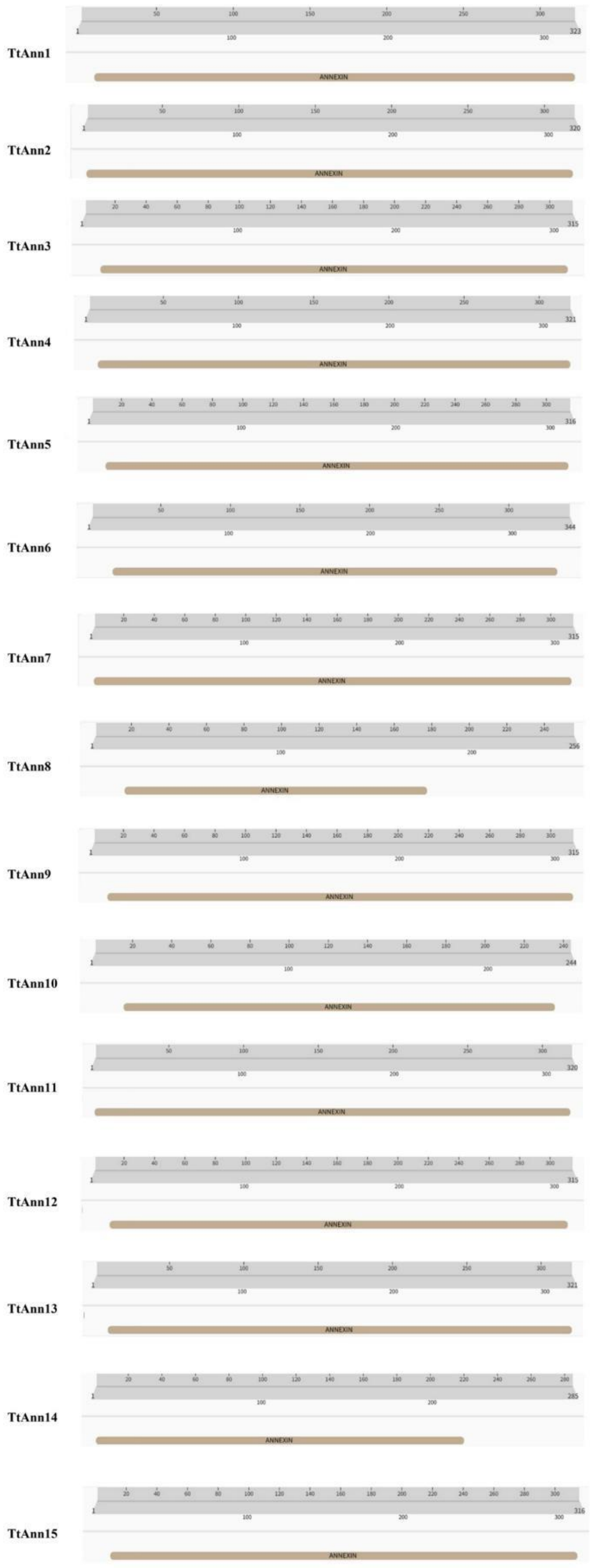

Figure S2 The pfam domain diagrams of 15 TtAnns identified on the InterPro website (http://pfam.xfam.org/).

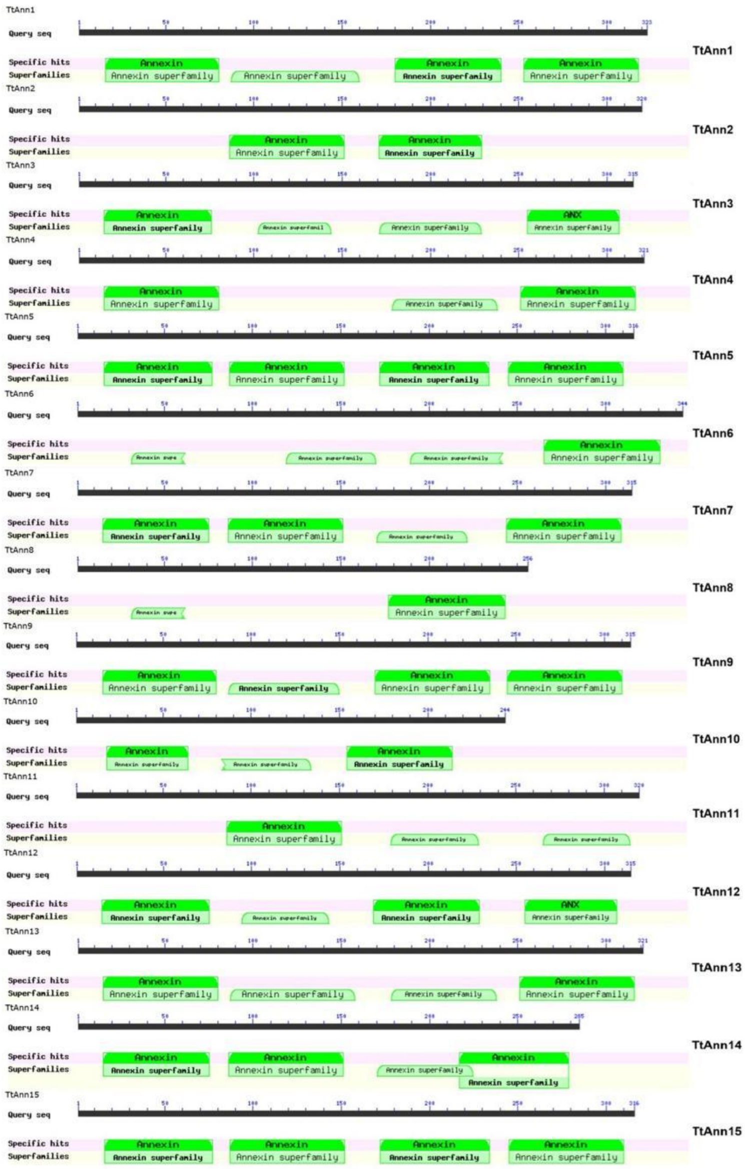

Supplement: Supplementary file 1 [file plants-15-02218-s001.zip › plants-4363568-Figures S1 and S2.pdf]
